# Emotional labor and burnout among nurses in Iran: core self-evaluations as mediator and moderator

**DOI:** 10.1186/s12960-024-00896-y

**Published:** 2024-02-09

**Authors:** Elham Saei, Soheil Sarshar, Raymond T. Lee

**Affiliations:** 1 Department of Psychology, Shahid Chamran University of Ahvaz, Ahvaz, Iran; 2 Department of Psychology, Allameh Tabataba’i University, Tehran, Iran; 3 Department of Business Administration, Asper School of Business, University of Manitoba, Winnipeg, Canada

**Keywords:** Nurse burnout, Core self-evaluations, Emotional labor, Surface acting, Deep acting, Pandemic

## Abstract

**Background:**

This study investigated the mediating and moderating impact of core self-evaluations in the path from emotional labor to burnout. Our hypothesized associations are based on Hobfoll (Rev Gen Psychol 6:307–24, 2002) conservation of resources theory.

**Method:**

Three hundred nurses from four hospitals in Abadan, Iran, were invited to participate in our study. Of the 300, 255 completed all sections and questions in our survey for an 85% response rate. The posited direct and indirect effects were evaluated with structural equation modeling and the interaction effects were evaluated with hierarchical moderated regression and simple regression slope plots.

**Result:**

Deep acting has indirect effects on burnout through core self-evaluations. Though unrelated to surface acting, core self-evaluations moderate its impact: under low core self-evaluations, surface acting is strongly related to emotional exhaustion and inversely related to personal accomplishment, whereas, under high core self-evaluations, surface acting is unrelated to these burnout dimensions.

**Conclusion:**

Our findings reveal the dual functions of CSE as a psychological resource and buffer to offset the interpersonal demands of patient care. Limitations, directions for future research, and practical implications are discussed.

*This above all: To thine own self be true*

* ~ Hamlet, Act 1, Scene III, line 78 *[1]

## Background

Research has documented that many nurses have been under distress due to excessive work demands, but especially pronounced during the pandemic of 2020–2021 [2, 3]. Galanis et al. [2] found that younger and less experienced nurses experienced higher burnout levels during COVID. Martin et al. [3] found that during the pandemic, registered nurses in the US, especially the younger and less experienced, endured heavy workloads and had unprecedented burnout levels. These factors have led to higher than usual turnover with the potential for further declines. Similarly, Murat et al. [4] found that nurses in Istanbul, who were younger and had fewer years of work experience, felt inadequately trained in nursing care and experienced heightened distress and burnout. Increased burnout levels were detected among nurses with positive COVID‐19 test results and did not wish to work voluntarily during the pandemic.

Lack of clinical experience and training are challenging factors, especially during patient care that requires emotional labor, which has been linked to nurse burnout [5]. More broadly, all healthcare professionals must be able to muster sufficient resources to either mitigate or prevent burnout, which may adversely impact mental and physical wellbeing, as well as job performance [6]. Our particular focus on nurses, however, is due to the necessity of providing patient comfort and reduce distress for which emotional labor is an integral part of demonstrating clinical care [5, 7]. We examine how core self-evaluations, as a personal resource [8], can mediate to offset the demands of emotional labor *and* moderate to buffer the negative influence of emotional labor on burnout.

The conservation of resources theory [9] explains the underlying mechanisms of the posited relationships [10]. According to this theory, when individual resources are threatened or depleted, these deficits lead to heightened anxiety and distress, thereby impacting physiological arousal and health states [8]. Experiencing high interpersonal demands and constraints may pose a threat to one’s self-image, leading to greater cortisol response and strain than other stressors [11].

The theory has been applied to examining how nurses respond to losses in four specific resources: objects, conditions, personal characteristics, and energy [12]. Prapanjaroensin et al.’s review found that perceived or actual loss of these resources led to increased burnout levels, which undermined work performance, including the overall quality of clinical care [12].

## Emotional labor and burnout

Self-regulatory efforts may lead to resource loss for two reasons. First, the inauthenticity of faked expressions reduces one’s self-worth and self-efficacy [13]. Second, suppressing emotions consumes energy, reflected in increased physiological arousal, higher glucose levels, and decreased motivation [11, 14]. Altering one’s feelings, thoughts, and behaviors through emotional faking and suppression leads to surface acting, which requires effort, consuming both cognitive and emotional resources that diminishes wellbeing [15].

While deep acting still requires energy to align feelings with displayed emotions, it does not require the same degree of self-regulatory effort as one expresses genuine feelings, hence, would not incur the same amount of resource loss as surface acting. A study of South Korean nurses found through path analysis that surface acting was positively associated with burnout through the stress of emotional labor, which was positively associated with burnout [5]. It further revealed that deep acting was not associated with any of the emotional labor-related stress, including burnout. We predict that:

H1: Surface acting would be associated with emotional exhaustion and depersonalization and inversely associated with personal accomplishment.

H2: Deep acting would not be associated with any of the three burnout dimensions.

## Core self-evaluations as mediator and moderator

Core self-evaluations are conceptualized as comprising four components: (a) self-esteem, (b) generalized self-efficacy, (c) locus of control and (d) emotional stability [16]. The evaluation of one’s own ability and values through core self-evaluations [17] is posited as a critical resource inversely associated with burnout [18]. It also has been linked to job engagement [19], and work motivation [20], leading to increased capability, positive self-concept, adaptation, and high self-confidence [21], which all have been associated with positive job attitudes and performance [22].

Core self-evaluations play a pivotal role between emotional labor and burnout. A study of nurses in Pakistan found this trait was associated with patient-oriented behaviors, defined as the enthusiasm of nurses to modify their service delivery based on their patients’ requirements and conditions [7]. Such behaviors encompass communication skills, courteousness, and mindfulness to the patients’ needs. Their findings suggest that expressing patient-oriented behaviors through emotional labor draws upon the mental and emotional resources of core self-evaluations to meet their demands.

The direct and indirect association between core self-evaluations and nurse burnout has been documented in other studies [23–26]. Taken together, the research of nurses suggests that core self-evaluations can facilitate and attenuate the negative influence of emotional labor, especially surface acting, on burnout.

Our study builds on this body of research on the intervening role of core self-evaluations [27, 28]. Based on the conservation of resources theory [8], core self-evaluations are considered to be an enduring, stable set of resources that increase nurses’ motivation and effort [21] to meet the interpersonal demands of service work and facilitates self-efficacy to reduce burnout [29].

The opportunity to express naturally and heartfelt emotions reinforces a nurse’s sense of personal authenticity and core self-evaluations [13]. In contrast, surface acting fosters dissonance of having to either display unfelt emotions or suppress undesirable feelings during clinical care [27, 29], which undermines authentic self-expression [13] and diminishes core self-evaluations. We predict that:

H3: Deep acting would be associated with core self-evaluations, and

H4: Surface acting would be inversely associated with core self-evaluations.

As stated above, core self-evaluations intervene in two ways: first, it may facilitate the path from emotional labor to burnout; second, it may modify their relations. As positive self-concept, adaptation, and high self-confidence represent psychological resources [21], the trait is posited to facilitate interpersonal resources by strengthening one’s sense of personal accomplishment while reducing emotional exhaustion.

Authentic self-expression through deep acting leads to increased self-efficacy as a form of self-evaluation [30]. Sheldon et al. [31] found that authenticity was strongly associated with mental wellbeing and role-specific satisfaction. Hence, as deep acting reinforces positive core self-evaluations, this trait also will ameliorate emotional exhaustion and depersonalization, while bolstering personal accomplishment. We predict that:

H5: Core self-evaluations would mediate between deep acting and the burnout dimensions. Specifically, deep acting would increase core self-evaluations, which in turn, would decrease emotional exhaustion and depersonalization and increases personal accomplishment.

**Fig. 1:**
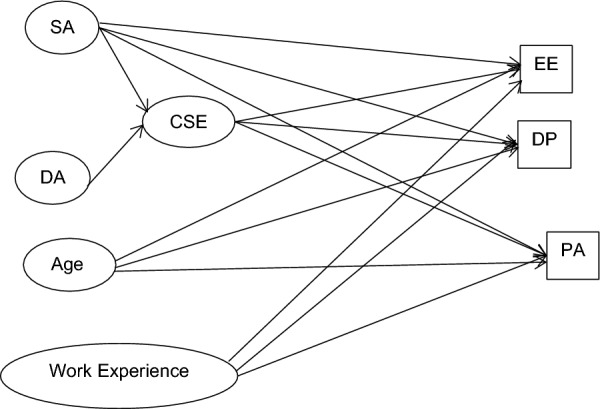
Predicted relations in structural model. *CSE* core self-evaluations, *SA* surface acting, *DA* deep acting, *EE* emotional exhaustion, *DP* depersonalization, *PA* personal accomplishment

Because core self-evaluations and surface acting are posited to be inversely related, no facilitation effect is proposed. Nevertheless, we explore whether the trait mediated for surface acting. Figure 1 diagrams the predicted structural relations.

Following the conservation of resources theory [8, 9] core self-evaluations will buffer the demands of engaging in surface acting, as well as reduce the dissonance of expressing unfelt emotions or hiding undesirable feelings when providing care [27, 29]. As noted above, the trait will influence patient-oriented behaviors [7], which in turn, requires emotional labor that consumes mental and emotional resources to satisfy patient demands. Baig et al. [7] found that core self-evaluations moderated the impact of employee motivation on patient***‐***oriented behavior. We posit that this path can be extended where core self-evaluations also buffer the impact of emotional labor on burnout.

Specifically, core self-evaluations will diminish the association between surface acting and burnout: compared to low levels of core self-evaluations, high levels of this trait will attenuate the surface acting → emotional exhaustion and depersonalization paths and also will weaken the surface acting → diminished personal accomplishment path. We predict that:

H6: Core self-evaluations would moderate the effect of surface acting on emotional exhaustion and depersonalization, such that the links are weaker as the trait increases from low to high levels, and on diminished personal accomplishment, such that the link is weaker as the trait increases from low to high levels.

Although not predicted, we also explore whether core self-evaluations moderate for deep acting.

## Methods

### Research setting and context

Our study was in Abadan, a port city with an oil refinery as the major industry near the Persian Gulf in southwest Iran. When research was undertaken in 2019, the pandemic had just started in Iran. While our study participants did not see COVID-diagnosed patients as they were not sent to the wards taking part in our study, the nurses nevertheless were uncertain on whether those in their care had contracted COVID-19. The Iranian government had not declared a pandemic at the time, but the hospital staff minimized close contact with patients and visitors, all the while experiencing heightened anxiety and distress.

### Participants and data collection

The study design was a single wave, cross-sectional self-report questionnaire survey containing validated measures where the nurses reported on their work experiences during the onset of the pandemic in 2019–2020. Using multi-stage sampling, four out of nine hospitals within Abadan were randomly chosen. Then a set of in-patient wards common across these four hospitals was identified: cardiology, internal medicine, gynecology, general surgery, and hemodialysis. These wards treated a diverse mix of patients requiring different forms of clinical care and amount of emotional labor from the nurses. Lastly, 300 nurses from across these five wards at each of the four hospitals were randomly invited to take part in our study (i.e., participants drawn from five wards x four hospitals = 20 different work units). For those who declined to participate before receiving the survey, we randomly invited the same number of replacement nurses drawn from the five wards to maintain the sample size of 300.

All demographic information in our study sample was collected from the survey. In the sample, 178 (70%) self-identified as female; 77 self-identified as male. The mean age was 29.35 years; the mean work experience was 4.64 years. The distribution of the education degree attained is as follows: diploma at 3.5%, associate at 1.2%, bachelor’s at 78.9%, Master’s at 4.7%, and doctorate at 11.7%. Compared to all nursing staff across the four hospitals chosen, which had a mean age of 31 years, and 71% being female, our sample was representative of the nurse population there.

We compared our demographic profile with those reported in other studies of nurse burnout in Iran. Shamsi et al. [32] reported that the age range of nurses in Iran was 35–38 years, which overlaps with our sample’s age range. Vahedian-Azimi et al. [33], reported that in their sample, the mean age was 29 years, 69% were female, and 74% had bachelor’s degrees. Hasanpoor et al. [34] reported that in their sample, 63% were female, and 63% had bachelor’s degrees. Maleki et al. [35], reported that in their sample, 78% wew female, and 85% had bachelor’s degrees. Sadeghi et al. [31] reported that in their sample, 60% were female, and 84% had a bachelor’s degrees. Jalali et al. [36] reported that in their sample, 75% were female, and 88% had bachelor’s degrees. Akbari et al. [37] reported that in their sample, 79% had bachelor’s degrees and 6% had Master’s degrees. Lastly, Fallah et al. [38] reported in their sample, 67% were female, and 40% had work experience between 1–5 years. Relative to the nurses sampled in these studies, our sample of nurses shared comparable demographic characteristics.

Following the hospital’s human resource management protocol, 5 days before data collection, we sent to nurses by SMS a brief message about our study and an invitation to complete the survey. The nurses who indicated a willingness to take part signed an informed consent form issued by the hospitals’ parent company and received additional information on the research, including the aim and variables, how the findings would be disseminated through a survey feedback report, and the parties responsible for protecting the confidentiality of the study data. Then 5 days after our SMS, the participants were sent a self-report survey questionnaire.

The participants were informed that they were free to opt out at any time they did not wish to continue in our study. As noted above, to replace the nurses who opted out, we randomly invited an equal number of nurses from the same wards to replace the drop-out participants. With the help of the hospital's human resources management, nurses who dropped out were temporarily reassigned to other wards where the research was not being conducted to minimize any contact with colleagues who were taking part until the data collection was completed in one week’s time.

### Study variables and measures

The variables evaluated in our six hypotheses are as follows: core self-evaluations; emotional labor dimensions of surface acting and deep acting; burnout dimensions of emotional exhaustion, depersonalization, and diminished personal accomplishment; age and work experience in nursing.

*Core self-evaluations* were measured by 12 items (e.g., “I am confident I get the success I deserve in life,” and, “I determine what will happen in my life.”) developed and validated by Judge et al.[39]. Each item has a five-point Likert-type response scale ranging from 1 (“strongly disagree”) to 5 (“strongly agree”).

*Emotional labor* taps the different forms of emotional regulation and expression during in-role service encounters [40]. The surface acting and deep acting dimensions are from the *Emotional Labour Scale*, developed and validated by Brotheridge and Lee [40, 41]. Surface acting has three items on the extent of faking (e.g., “Showing emotions that I don't feel”) and suppression (e.g., “Hide my true feelings about a situation”). Deep acting has three items on the extent feelings were modified to align with display rules (e.g., “Try to actually experience the emotions that I must show”)**.** Each item has a five-point Likert-type response scale ranging from 1 (“rarely or never”) to 5 (“always or most often”). Surface acting and deep acting have been found to be related [41], but differentially related to the dimensions of burnout [6, 13].

The *Maslach Burnout Inventory* was used to assess nurse burnout. This measure comprised emotional exhaustion with nine items, depersonalization with five items, and personal accomplishment with eight items [42]. Emotional exhaustion occurs when one’s emotional energy is depleted. Depersonalization is when one feels indifference to or estranged from recipients, which for nurses were their patients. Personal accomplishment is diminished when one lacks a sense of efficacy to perform well. Each item has a seven-point Likert-type response scale ranging from 0 = (“never”), to 6 = (“every day”). The dimensions have been found to be independent factors but inter-related. [42, 43]

All measures were translated to Persian and back translated to English to check for accuracy.

### Control variables

Brewer and Shapard’s [44] found small inverse correlations between age and work experience with EE for employees in certain fields. Galanis et al. [2] revealed that younger age is a risk factor in nurses. The evidence that burnout decreases with age may be due to the accumulation of work experience and resources for coping with distress [45]. Based on these findings, we specified age and work experience as exogenous predictors of each burnout dimension.

### Data analyses

To check for common method variance, we compared a six-factor measurement model with the surface acting items on factor one, deep acting items on factor two, core self-evaluations items on factor three, emotional exhaustion items on factor four, depersonalization items on factor five, and personal accomplishment items on factor six, against a one-factor model where all items of these variables were specified to load on a common method factor.

Instead of individual items, composite indicators were used for the measurement models, as retaining the individual items would likely have yielded a poorer fit. Items were combined to form multiple indicators for core self-evaluations and the burnout dimensions, following the procedure used by Lee and Ashforth [43]. A one-factor model was fit to the items for each variable. Items with the highest and lowest loadings were combined first, items with the next highest and lowest loadings were combined next, and so on until all items of a variable had been assigned to one of the composite indicators.

The direct and indirect (i.e., mediated) effects were evaluated through structural equation modeling with *SPSS*-AMOS 22. To construct the 95% confidence interval for each indirect effect, its standard error (SE) was estimated through bootstrapping [46].

Due to the number of parameters estimated and possible multicollinearity with adding the interaction paths to our predicted models, we used ordinary-least-squares hierarchical moderated regression evaluated with the *R*^2^ difference test. For each significant interaction, the simple regression slopes of low (one *SD* below the mean) and high (one *SD* above the mean) core self-evaluations levels were plotted.

## Results

### Retained sample

After the survey was administered, 280 out of 300 study participants returned their completed questionnaires. Of these, 25 were excluded from subsequent analyses either because of missing information (*n* = 20) or had extreme response values (*n* = 5), identified as outlier observations (see explanation below). Thus, our *retained sample size* is 255, yielding an 85% response rate (i.e., *N* = 255 usable returns out of 300 administered surveys).

### Multivariate normality

Structural equation modeling requires certain underlying assumptions be met to ensure accurate inference. A fundamental assumption is that observations are drawn from a continuous and multivariate normal population, especially for maximum likelihood estimation of structural relations. If the data follow a continuous and multivariate normal distribution, then maximum likelihood solutions will yield unbiased parameter estimates. In contrast, non-normality leads to an overestimation of the *χ*^2^ fit ratio, potentially leading to false rejection of the model as a whole, where the underestimation of standard errors lead to inflated statistics and possibly erroneous attributions of the significance of relationships in the model [47]. The overestimation of the *χ*^2^ ratio is directly related to the degrees of freedom in the hypothesized models [48]. However, this fit index will not be overly inflated by slight non-normality, where the parameter estimates will not be severely biased [49]. Indeed, standard errors may be even underestimated relative to the standard deviations of the parameter estimates.

In AMOS 22, multivariate normality is measured by Mardia’s multivariate elasticity [47]. Outliers are indicated by their Mahalanobis distances, which represent the standard unit squared distances of an observation vector from the sample mean vector of the variables. The greater the distance, the more the observations contribute to the multivariate elasticity of Mardia and departure from multivariate normality. Removing an outlier may reduce Mardia’s multivariate skewness. Outliers can be sequentially excluded until the desired multivariate kurtosis index is reached. The advantage of removing outliers over transforming the data to achieve normality is that it preserves the linearity assumption. The disadvantage of removing outliers is the loss of observations, hence, fewer degrees of freedom and statistical power.

We performed the multivariate normality test and found that the critical *t*-ratio is 3.879, which is greater than 1.96 and indicative of non-normality. When the critical *t*-ratio is *smaller* than 1.96, this indicates that the coefficient of multivariate kurtosis is not significantly different from zero (i.e., normally distributed) [47]. Thus, we excluded observations with large Mahalanobis outliers to reduce multivariate skewness. We continued excluding observations step-by-step until reaching close to multivariate normality. Five observations were identified as major outliers as their Mahalanobis distances were much larger than the distances of others. Excluding them decreased both univariate and multivariate non-normality, indicating that the five outliers heavily skewed the data’s distribution. The marginal and cumulative contributions of the remaining outliers to non-normality were small. Excluding them decreased both univariate and multivariate non-normality, indicating that the five outliers heavily skewed the data’s distribution.

But there remained this question if multivariate normality can be achieved by excluding the outliers, to what extent would statistical power be permitted to be reduced due to a smaller sample size [48]? So, we determined the specific level of multivariate non-normality that would yield a relatively unbiased estimation of all parameter estimates, standard errors, and Chi-square (henceforth *χ*^2^) fit ratios. Therefore, we concluded that only these five observations needed to be excluded from our analyses of structural relations without sacrificing power.

### Evaluation of models and hypotheses

The measurement model comparisons reveal that the six-factor model [*χ*^2^ = 437.96, d*f* = 120, *χ*^2^/d*f* = 3.82; NFI = 0.97; CFI = 0.95; RMSEA = 0.07] was superior in fit to the one-factor model [*χ*^*2*^ = 4635.36; d*f* = 740; *χ*^2^/d*f* = 6.26; NFI = 0.81; CFI = 0.76; RMSEA = 0.12], with their *X*^*2*^ (d*f*) difference of 4197.40 (620) significant at the 0.001 level.

Table 1 shows the means, standard deviations, correlations, and Cronbach *α* estimates of the internal consistency reliability of measures used in our study.

**Table 1:** Means, standard deviations, reliability estimates, and correlations

Variable	*M*	SD	1	2	3	4	5	6
1. CSE	38.67	5.46	(0.85)					
2. SA	11.84	4.01	− 0.07	(0.98)				
3. DA	11.94	5.18	0.24^**^	− 0.07	(0.94)			
4. EE	16.41	2.28	− 0.17^*^	0.52^**^	− 0.01	(0.84)		
5. DP	9.39	5.39	− 0.15^*^	0.50^**^	− 0.01	0.71^**^	(0.82)	
6. PA	26.98	4.33	0.36^**^	− 0.10	0.14^*^	− 0.23^**^	− 0.12^*^	(0.85)
7. Age	29.35	5.29	− 0.19^*^	0.07	− 0.10	0.19^*^	0.04	0.04
8. Exp	4.64	4.69	− 0.46^**^	− 0.02	0.02	0.16^**^	0.02	0.04

*N* = 255, after listwise deletion of missing data and outlier observations

Cronbach *α* estimates are in parentheses

*CSE* core self-evaluations, *SA* surface acting, *DA* deep acting, *EE* emotional exhaustion, *DP* depersonalization, *PA* personal accomplishment, *Exp.* work experience

*p < 0.05; **p < 0.001

We first evaluated the predicted paths with age and work experience as control variables. The results indicated that both are weakly positively related only to emotional exhaustion, which is the opposite direction of what Brewer and Shapard [44] found. Moreover, the two did not significantly alter the other structural relations compared to when they are not in the model. All subsequent analyses excluded the control variables in the trimmed structural relations model.

The trimmed model’s fit indices are as follows: *χ*^2^ (129) = 254.81; normed fit index (NFI) = 0.95; comparative fit index (CFI) = 0.97; root mean square error of approximation (RMSEA) = 0.05. To meet the criteria for adequate fit [50], the *χ*^2^/d*f* ratio should be less than five, with ours at 1.98; the NFI and CFI should each be 0.95 or greater, with both of ours meeting the cutoff value; and the RMSEA should be 0.05 or less, with ours at the cutoff value.

Table 2 shows the direct effects. For H1, which posits that surface acting to be related to burnout, the results indicate the effects to be significant for emotional exhaustion (*β* = 0.30, *p* < 0.05), depersonalization (*β* = 0.44, *p* < 0.05) and personal accomplishment (*β* = − 0.15, *p* < 0.05). For H2, which posits that deep acting is unrelated to burnout, the results indicate the effects to be non-significant for emotional exhaustion (*β* = − 0.10, ns), DP (*β* = − 0.09, ns), and personal accomplishment (*β* = 0.06, ns). For H3, which posits that deep acting to be related to core self-evaluations, the results indicate the effect to be significant (*β* = 0.25, *p* < 0.01). For H4, which posits that surface acting to be negatively related to core self-evaluations, the results indicate the effect to be non-significant (*β* = 0.04, ns). This also means that it would not mediate between surface acting and burnout. In sum, H1, H2, and H3 are supported; H4 is not supported.

**Table 2:** Direct effects, standard errors, and significance tests

Specified path	Direct effect	*SE*	Critical *t*-ratio	*p*
^SA→CSE^	0.04	0.18	0.55	0.583
^SA→EE^	0.30	0.33	2.17	0.001
^SA→DP^	0.44	0.16	5.60	0.001
^SA→PA^	− 0.15	0.23	7.28	0.030
^DA→CSE^	0.25	0.13	3.33	0.001
^DA→EE^	− 0.10	0.25	1.34	0.158
^DA→DP^	− 0.09	0.12	0.79	0.181
^DA→PA^	0.06	0.18	2.16	0.427
^CSE→EE^	− 0.15	0.14	2.16	0.030
^CSE→DP^	− 0.14	0.07	2.04	0.041
^CSE→PA^	0.39	0.10	5.47	0.001

*CSE* core self-evaluations, *SA* surface acting, *DA* deep acting, *EE* emotional exhaustion, *DP* depersonalization, *PA* personal accomplishment

Table 3 shows the indirect effects path coefficients. For H5, which posits that core self-evaluations to mediate deep acting and the burnout dimensions, the results indicate the indirect effects to be significant for emotional exhaustion (*β* = 0.14, *p* < 0.05), depersonalization (*β* = 0.09, *p* < 0.05), and personal accomplishment (*β* = 0.12, *p* < 0.05). Thus, H5 is supported.

**Table 3:** Indirect effects and confidence intervals

Specified path	Indirect effect	95% confidence interval		*p*
		Upper	Lower	
^DA→CSE→EE^	0.14	0.16	0.05	0.035
^DA→CSE→DP^	0.09	0.09	0.01	0.029
^DA→CSE→PA^	0.12	0.08	0.01	0.030

*CSE* core self-evaluations, *DA* deep acting, *EE* emotional exhaustion, *DP* depersonalization, *PA* personal accomplishment

**Fig. 2:**
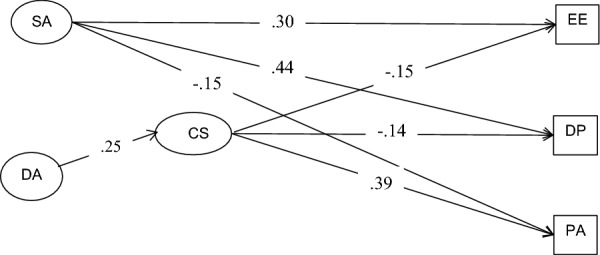
Significant paths in the trimmed structural model. *CSE* core self-evaluations, *SA* surface acting, *DA* deep acting, *EE* emotional exhaustion, *DP* depersonalization, *PA* personal accomplishment

Figure 2 diagrams all significant direct and indirect paths in the trimmed structural relations model.

For H6, which posits that core self-evaluations moderate between emotional labor and burnout, Table 4 shows the results of the hierarchical moderated regression models. The interactions with surface acting are significant for emotional exhaustion (*R*^2^ change = 0.02,* p* < 0.05) and personal accomplishment (*R*^2^ change = 0.03, *p* < 0.05), lending support for H6. Although not predicted, the interactions with deep acting are significant for emotional exhaustion (*R*^2^ change = 0.05, *p* < 0.001) and depersonalization (*R*^2^ change = 0.06, *p* < 0.001).

**Table 4:** Hierarchical moderated regressions

Burnout	Model	*R*^2^	Δ*R*^2^	*t*-ratio	*p*
EE	SA	0.54^**^			
	CSA				
	SAxCS	0.56^**^	0.02^*^	2.55	0.011
DP	SA	0.51^**^			
	CSA				
	SAxCS	0.52^**^	0.01	1.05	0.293
PA	SA	0.38^**^			
	CSA				
	SAxCS	0.41^**^	0.03^*^	2.61	0.010
EE	DA	0.18^*^			
	CSA				
	DAxCSE	0.23^**^	0.05^**^	2.39	0.018
DP	DA	0.15^*^			
	CSA				
	DAxCSE	0.21^**^	0.06^**^	2.41	0.017
PA	DA	0.37^**^			
	CSA				
	DAxCSE	0.37^**^	0.00	0.15	0.882

*CSE* core self-evaluations, *SA* surface acting, *DA* deep acting, *EE* emotional exhaustion, *DP* depersonalization, *PA* personal accomplishment

Δ*R*^2^: change in explained variance with the interaction term entered

*p < 0.05; **p < 0.001

**Fig. 3:**
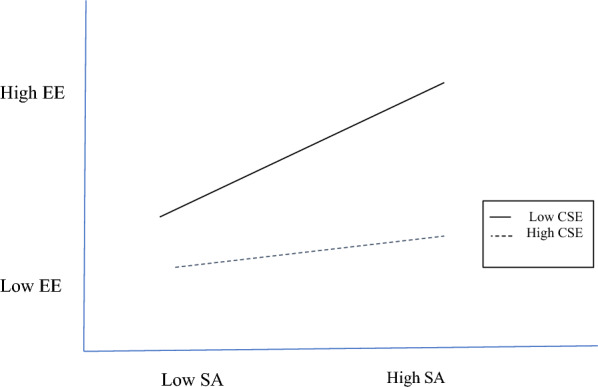
Core self-evaluations moderating surface acting and emotional exhaustion. *CSE* core self-evaluations, *SA* surface acting, *EE* emotional exhaustion

Figure 3 plots the surface acting and emotional exhaustion interaction. With low core self-evaluations, whether surface acting was performed yields a significant difference (*β* = 0.30, *p* < 0.001) in emotional exhaustion levels, whereas with high core self-evaluations, whether surface acting was performed yields no significant difference (*β* = 0.03, ns) in emotional exhaustion levels. The lowest level is with low surface acting and high core self-evaluations; the highest emotional exhaustion level is with high surface acting and low core self-evaluations.

**Fig. 4:**
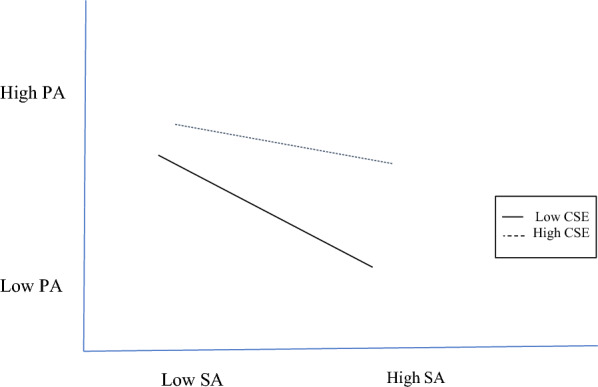
Core self-evaluations moderating surface acting and personal accomplishment. *CSE* core self-evaluations, *SA* surface acting, *PA* personal accomplishment

Figure 4 plots the surface acting and personal accomplishment interaction. With high core self-evaluations, whether surface acting was performed yields no significant difference (*β* = − 0.05, ns) in personal accomplishment levels, whereas with low core self-evaluations, whether surface acting was performed yields a significant difference (*β* = − 0.22, *p* < 0.001) in personal accomplishment levels. The highest personal accomplishment level is with low surface acting and high core self-evaluations; the lowest personal accomplishment level is with high surface acting and low core self-evaluations.

**Fig. 5:**
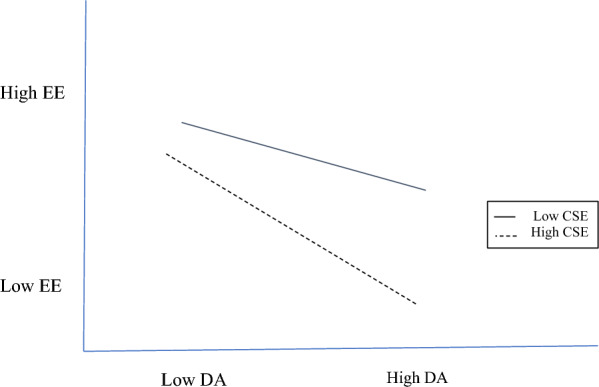
Core self-evaluations moderating deep acting and emotional exhaustion. *CSE* core self-evaluations, *DA* deep acting, *EE* emotional exhaustion

Figure 5 plots the deep acting and emotional exhaustion interaction. With low core self-evaluations, whether deep acting was performed yields no significant difference (*β* = − 0.06, ns) in emotional exhaustion levels, whereas with high core self-evaluations, whether deep acting was performed yields a significant difference (*β* = − 0.20, *p* < 0.05) in emotional exhaustion levels. The lowest emotional exhaustion level is with high deep acting and high core self-evaluations; the highest emotional exhaustion level is with low deep acting and low core self-evaluations.

**Fig. 6:**
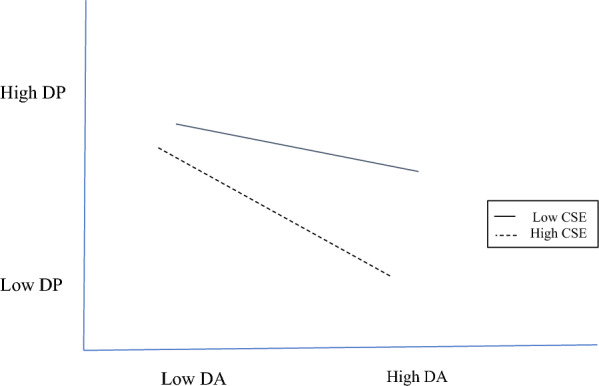
Core self-evaluations moderating deep acting and depersonalization. *CSE* core self-evaluations, *DA* deep acting, *DP* depersonalization

Figure 6 plots the deep acting and depersonalization interaction. With low core self-evaluations, whether deep acting was performed yields no significant difference (*β* = − 0.01*, ns*) in depersonalization levels, but with high core self-evaluations, whether deep acting was performed yields a significant difference (*β* = − 0.18, *p* < 0.05) in depersonalization levels. The lowest depersonalization level is with high deep acting and high core self-evaluations; the highest depersonalization level is with low deep acting and low core self-evaluations.

The non-significant correlation of surface acting and deep acting shown in Table 1 (*r* = − 0.07, ns), suggests that the display of one did not necessarily coincide with the other, indicating that core self-evaluations’ moderating influence on each of the emotional labor dimensions to operate independently of one another. When the need for surface acting was low, higher core self-evaluations were strongly associated with heightened self-efficacy in role performance.

## Discussion

The most distinct contribution of our study is documenting core self-evaluations’ dual functions. This duality is reflected in Polonius’ advice to Laertes as quoted [51] in our paper’s opening. His guidance has two pertinent meanings [1]: the first is to be forthright in one’s interactions: Laertes’ dealings with business acquaintances, and in our study, nurses’ sharing heartfelt emotions during clinical care. The second is to act in ways that benefit oneself: Laertes advised to spend his financial resources prudently, and in our study, nurses drawing upon their personal resources to ameliorate the adverse influence of surface acting.

Our findings reveal that emotional demands were offset by core self-evaluations in these two distinct ways. The first is to facilitate the path from deep acting to reduced emotional strain and detachment and increased self-efficacy [30]. Similarly, a study of nurses in China found that social support increased core self-evaluations, which in turn, facilitated greater job involvement and bolstered self-appraisal [52]. The second is to dampen the harmful impact of surface acting on wellbeing [15]. Core self-evaluations engendered positive feelings and motivated the nurses to show emotions beneficial to their patients as well as to themselves. Social support from peers and patients would have further bolstered positive self-appraisal [52].

As noted above, core self-evaluations represent a fundamental trait closely tied in with self-confidence [21], motivation and effort [20], job engagement [19], and job success [22]. It also is a psychological resource for coping with stressful events [8, 11]. Our findings reveal the central role of this trait in bridging interpersonal demands with nurses’ wellbeing [10]. Other studies of nurses have examined alternative intervening variables One found that surface acting was associated with dissonance, which in turn, increased nurse burnout [27]. Another found that leader–member exchange moderated the relationship between emotional labor and nurse burnout [28]. In contrast, we found deep acting to be associated with increased core self-evaluations, which in turn, reduced burnout.

As a mediator, caregiving through emotional communications bolstered nurses’ sense of self-worth (“doing the right thing for patients”), which in turn, enhanced their own wellbeing. As a moderator, nurses responded to the interpersonal demands in ways that benefited their own self-interests (e.g., adopting positive or optimistic outlook, taking control during stressful encounters), which buffered the negative impact of emotional labor.

Core self-evaluations are more strongly associated with personal accomplishment (Table 2: direct effect = 0.39) than with either emotional exhaustion (direct effect = − 0.15) or depersonalization (direct effect = − 0.14). Personal accomplishment represents an aspect of self-efficacy tied to adjustment under stressful conditions [53]. Self-efficacy is linked to self-assessment of performance and the desire to be in control [23]. Lee and Ashforth also found this burnout dimension to be associated with control as a coping response and self-appraisal of job performance among social work supervisors and managers [43]. As our data were gathered during the pandemic’s initial wave, the uncertainty and resource constraints likely led to increased burnout [2].

Sincerity with others is reflected in authentic self-expression that emerges through deep acting and is associated with reduced emotional exhaustion and increased personal accomplishment [13]. As Sheldon et al. [31] noted, authenticity is based on how much of one’s behaviors are authored by the self. Thus, to the extent that core self-evaluations cultivate genuine displays, deep acting reinforces and sustains such candor.

Acting for self-gain is done by harnessing one’s core self-evaluations too. As a psychological buffer for the nurses in our study, positive outlooks reduced the adverse influence of surface acting on emotional exhaustion (Fig. 3), while personal accomplishment is at its peak when those with high core self-evaluations infrequently show surface acting (Fig. 4). A surprising finding is that the trait also moderates deep acting → emotional exhaustion and depersonalization, where the combination of positive outlooks and frequent show of deep acting is associated with lower emotional exhaustion (Fig. 5) and depersonalization (Fig. 6). For nurses, genuine expressions reduced or even neutralized dissonance [29], and allowed for rewarding relationships to develop [13].

Ideally, surface acting can be averted. In extreme events, however, the workplace’s display rules often require faking and suppression of emotions [54]. Under such stressful situations, caregivers with positive core self-evaluations may draw upon their personal resources [9] to adhere to the display rules when engaging in surface acting.

The following videos underscore core self-evaluations’ importance in service encounters. The first shows a traumatized worker using surface acting while collecting toll: https://www.youtube.com/watch?v=RuqR7iL8b3E [55]. The second shows another worker using deep acting while advising a driver stopped at a toll gate: https://www.youtube.com/watch?v=hAD9QlF_74Y [56]. These examples find parallels with nurses providing clinical care. Possessing high core self-evaluations lead to positive feelings and invigorates them [21], which is closely tied in with authentic expressions [57]. A positive reinforcement loop is generated where deep acting builds up self-mastery [53] and control [14, 30], leading to increased patient satisfaction and outcomes [58, 59].

Since our data were collected at the pandemic’s onset in Iran, we compared with other studies of nurse burnout during this period. In contrast to these findings [2–4], we found that age and work experience are *not* strongly associated with the burnout dimensions, as shown in Table 1. A surprising finding is that core self-evaluations are *negatively* associated with age (− 0.19) and work experience (− 0.46), also shown in Table 1. The nurses’ optimistic outlook, sense of control and self-efficacy were put to the test right from the pandemic’s start, especially among the more seasoned caregivers, which would have severely drained their personal resources [8, 11]. We posit that due in part to the pandemic, core self-evaluations played an especially *crucial role* in mitigating and buffering the negative impact of surface acting on emotional exhaustion and depersonalization.

### Limitations and future research

Several study limitations are worth noting. First, based on the above discussion of core self-evaluations as a critical set of resources, the obvious question is whether the trait’s mediating and moderating functions would have been comparable in our sample of nurses during the *pre-* and *post*-*pandemic stages*. A repeated-measures design over three periods (before, during and after) would have allowed us to evaluate the temporal robustness of core self-evaluations’ influence.

Second, the nurses invited to participate in our study were free to accept or not to accept participation. The pattern of responses of those who accepted may not have been the same as those who declined. Given the high response rate, however, any self-selection bias would likely be minimal.

Third, each of the participants was the sole information source of all study variables, increasing the likelihood of common method bias. However, it would not explain the differential relations found, particularly in the indirect effects for deep acting but not for surface acting, and how core self-evaluations moderated in four of the six emotional labor → burnout paths. Future studies could have coworkers and supervisors observe and rate the interactions of nurses [54]. Such performance ratings, along with objective behavioral and performance measures (e.g., tardiness, absenteeism, and error rates in medicating patients), may then be linked to symptoms of nurse burnout.

Last, the generalizability across hospitals and wards, both within Iran and to other nations, needs to be determined. While studies of emotional labor and nurse burnout abound [45], the extent to which this link extends to other contexts remains to be evaluated. For example, our study of nurses found the direct paths of surface acting → all burnout dimensions to be significant, whereas the direct paths of deep acting → all burnout dimensions not to be (Table 2). The broader question is whether our pattern of findings would be distinct or extend to caregivers across a wider range of caregiving settings.

### Practical implications

Given that high levels of core self-evaluations generate positive feelings and outlook, an obvious question is how nurses can effectively apply this critical trait. Hospital administrators and managers can foster a supportive work climate, initiate job enrichment, and empower nurses to craft their tasks to reduce burnout [60]. By taking ownership and pride in clinical care, nurses become more responsive to patients’ needs while still adhering to the spirit of the workplace’s display rules, much like the beaming toll cashier [56]. Encouraging deep acting reinforces self-mastery and professional efficacy [30, 53], even when unpleasant tasks are carried out, such as breaking bad news [61], and nurses reaching out to apathetic, distraught, or hostile patients [45, 59].

Among the nurses in our study, caring for others begins with self-care, especially when constraints and uncertainties are at their peak in troubled times [2]. Many nurses are able to muster coping resources on their own [10], while others are more reliant on workplace and patient support to bolster their self-confidence and motivation to meet the challenges of clinical care [60].

## Conclusion

Our findings suggest that core self-evaluations are resource linchpins in the duo capacity as mediator and moderator for nurses in the midst of the pandemic. Managing the demands of emotional labor through this trait will enhance clinical care and wellbeing for both patients and caregivers. Hospital leaders may contribute to empowering their nurses to take an optimistic outlook and bolster self-confidence, while simultaneously advancing a supportive workplace during and after this period.

## Data Availability

The datasets generated during and/or analyzed during the current study are not publicly available due to stipulations from the regional health and hospital authorities from where the data were collected but are available from the corresponding author on reasonable request.
